# A Challenge to Aging Society by microRNA in Extracellular Vesicles: microRNA in Extracellular Vesicles as Promising Biomarkers and Novel Therapeutic Targets in Multiple Myeloma

**DOI:** 10.3390/jcm7030055

**Published:** 2018-03-12

**Authors:** Tomofumi Yamamoto, Nobuyoshi Kosaka, Yutaka Hattori, Takahiro Ochiya

**Affiliations:** 1 Division of Molecular and Cellular Medicine, National Cancer Center Research Institute, 5-1-1 Tsukiji, Chuo-ku, Tokyo 104-0045, Japan; tomofumi-0321@keio.jp; 2 Clinical Physiology and Therapeutics, Keio University Faculty of Pharmacy, 1-5-30 Shibakoen, Minato-ku, Tokyo 105-0011, Japan; hattori-yt@pha.keio.ac.jp

**Keywords:** extracellular vesicles, exosome, microRNA, multiple myeloma, biomarker

## Abstract

Multiple myeloma (MM) is a malignancy of terminally differentiated plasma cells and is the second most common hematological cancer. MM frequently occurs in the elderly population with the median age as the middle sixties. Over the last 10 years, the prognosis of MM has been dramatically improved by new therapeutic drugs; however, MM is still incurable. The pathogenesis of MM is still unclear, thus greater understanding of the molecular mechanisms of MM malignancy is desirable. Recently, microRNAs (miRNAs) were shown to modulate the expression of genes critical for MM pathogenesis. In addition, miRNAs are secreted via extracellular vesicles (EVs), which are released from various cell types including MM cells, and these miRNAs are involved in multiple types of cell-cell interactions, which lead to the malignancy of MM. In this review, we summarize the current knowledge regarding the role of miRNA secretion via EVs and of EVs themselves in MM development. We also discuss the potential clinical applications of EVs as promising biomarkers and new therapeutic targets for improving the outcome of MM, resulting in a brighter future for aging societies.

## 1. Introduction

Extracellular vesicles (EVs) include exosomes, microvesicles and apoptotic bodies, and so on. They are secreted by almost all cell types and are classified by their origin, size, and properties [1,2]. Exosomes are small EVs of approximately 100 nm and are derived from the intracellular endosomal component. Exosomes are initially formed by a process of inward budding in early endosomes to form multivesicular bodies [3,4]. Exosomal markers include members of the tetraspanin family (CD9, CD63, CD81), members of the endosomal-sorting complex required for transport (TSG101, Alix), heat shock proteins (Hsp60, Hsp70, Hsp90), and Rab proteins (Rab27 A/B) [5,6]. Microvesicles (MVs) are larger than exosomes (approximately 100–1000 nm) [7]. They are composed of some lipid components and are directly shed or bud from plasma membranes. Although EVs were initially considered as cell waste, containing unwanted proteins and biomolecules [8], they were reported in 2007 to contain mRNA as well as microRNAs (miRNAs) [9]. In 2010, several groups demonstrated that miRNAs in EVs can be transferred to immune cells, cancer cells, and endothelial cells, and that these miRNAs have functions in the recipient cells [10,11,12]. Therefore, miRNAs in EVs represent a new form of intercellular communication.

**Figure 1:**
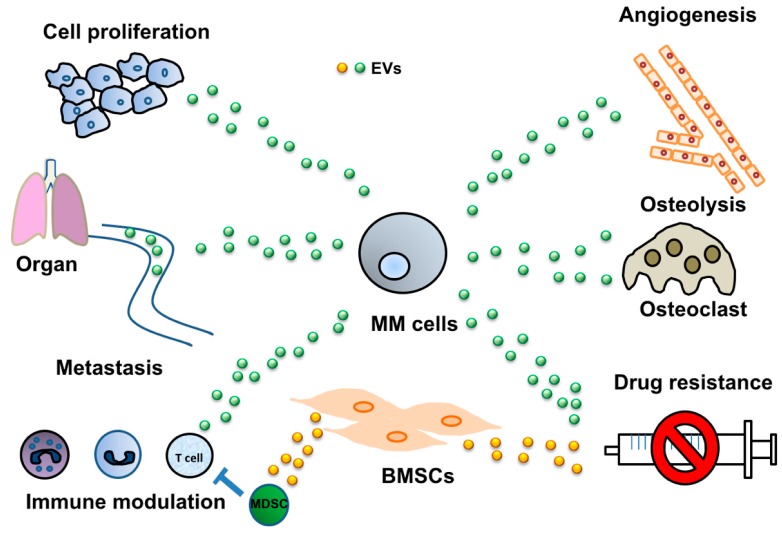
Influence of extracellular vesicles (EVs) on the multiple myeloma (MM) microenvironment. EVs are associated with various alterations in the MM microenvironment including angiogenesis, activation of osteolysis, immune system modulation, and induction of drug resistance. MM cells alter the surrounding microenvironment via EVs for their survival.

Over the last decade, accumulating evidence has shown that EVs can bridge cancer and normal cells. EVs transfer their components from one cell to another, including miRNAs, DNA, and proteins [13]. Tumor-derived EVs target various cell types to modify the tumor microenvironment, which supports the growth of tumor cells by inducing angiogenesis, tumor cell migration and metastasis, immune response modulation, and drug resistance. In this review, we summarize the current knowledge of the role of miRNA secretion via EVs in multiple myeloma (MM) development (Figure 1). Before describing the role of EVs in the progression of MM, several examples regarding EV-mediated cancer development will be introduced. Then, the potential clinical applications of EVs as promising biomarkers and new therapeutic targets for improving the outcome of MM will be discussed. We suggest that utilization of EVs for medicine will provide many benefits for aging societies.

## 2. Cancer-Derived EVs Mediate Various Cell-Cell Interactions

### 2.1. Induction of Angiogenesis via Cancer-Derived EVs

Because of the low blood flow, tumors become hypoxic and thus have low tissue oxygenation regions. Under hypoxic conditions, cancer cells undergo genetic alterations that allow them to survive in such an environment [14]. Under the hypoxic conditions in tumors, angiogenesis is crucial for the survival of cancer cells, and hypoxia also leads to a change in the cargo within EVs which induces the stress-relieving processes, like angiogenesis in the close or distant microenvironment [15]. Hypoxia-inducible factor-1 (HIF-1) promotes the production of angiogenesis-related genes, such as vascular endothelial growth factor. Abnormal angiogenesis in tumors is considered a major factor in cancer proliferation, therapy resistance, and metastasis. Communication between cancer and endothelial cells via miRNAs in EVs is associated with angiogenetic activity in tumors [15,16,17,18,19,20,21]. For instance, miR-23a in EVs directly suppresses its target prolyl hydroxylases 1 and 2, leading to the accumulation of HIF-1 α in the endothelial cells [20]. miR-23a also inhibits tight junction protein Zonula occludens-1, increasing vascular permeability and cancer transendothelial migration. Furthermore, miR-210, which is described as the major hypoxia-associated miRNA [22], in EVs contributes to tumor angiogenesis [15,19]. In summary, miRNAs in EVs alter the cancer microenvironment by modulating angiogenesis under hypoxic conditions.

Tumor-derived EVs can also affect distant endothelial cells [23]. Cancer cells migrate from a primary tumor to a secondary target organ via a progressive cascade of events, including microenvironmental remodeling processes at each stage of disease progression [24]. Endothelial cells are involved in the formation of a pre-metastatic niche, and cancer-derived EVs promote the alteration of distant endothelial cells [25,26]. EVs derived from a brain metastatic breast cancer cell line, which contains miR-181c, induce destruction of the blood-brain barrier [27]. miR-181c negatively regulates 3-phosphoinositide-dependent protein kinase-1, and causes degradation of phosphorylated cofilin and severing of actin filaments. Tumor-initiating cells expressing the mesenchymal stem cell marker CD105 in human renal cell carcinoma release EVs, triggering angiogenesis and promoting the formation of a pre-metastatic niche [28]. The authors found 82 miRNAs in the renal cell carcinoma-derived CD105+ EVs. Of these, 33 miRNAs had significantly lower levels and 24 miRNAs had significantly higher levels than in CD105- EVs.

As in solid tumors, EVs derived from hematological tumor cells, such as MM cells, also contribute to angiogenesis. EVs from murine MM carrying multiple angiogenesis-related proteins enhance angiogenesis and directly promote endothelial cell growth [29]. Several pathways, such as signal transducer and activator of transcription 3 (STAT3), c-Jun *N*-terminal kinase, and p53, are modulated by the EVs from MM in endothelial and bone marrow stromal cells (BMSCs). Moreover, heparan sulfate proteoglycans function as internalizing receptors of cancer cell-derived EVs, and their modification through antitumor action inhibits MM cell growth and angiogenesis via disruption of the heparanase/syndecan-1 (CD138) axis [30]. EVs derived from MM cells can affect various functions in mesenchymal stem cells (MSCs) via transferring miRNAs, such as miR-21 and miR-146a, which have been demonstrated to regulate MSC proliferation and transformation [31]. MM cells increase miR146a, which promote cytokine secretion, in MSC, resulting in the promotion of growth and migration in MM cells [32]. Angiogenesis in MM is considered an important factor in MM progression. As described above, MM-derived EVs have been associated with many angiogenesis pathways, not only in the microenvironment near cancer cells, but also in distant organs. Thus, understanding the involvement of EVs in angiogenesis is key to preventing the progression of MM.

### 2.2. Modulation of the Immune System by EVs Derived from MM

In cancer immunology, EVs function as both immune suppressors and promoters. During cancer development, cancer cells escape from immune systems [33]. Immune cells, such as macrophages, natural killer (NK) cells, T lymphocytes, and B lymphocytes, interact with cancer cells and regulate tumorigenesis and progression. Immune modulation via EVs contributes to tumor progression [34,35,36,37,38,39,40]. Cancer-derived EVs have immunosuppressive effects, and immune dysfunction mediated by EVs can help cancer progression and metastasis. In addition, tumor-derived EVs can increase cytokine production by myeloid-derived suppressor cells (MDSCs), which can suppress T-cell activation [37]. Furthermore, heat shock protein 72 on tumor-derived EVs enhances STAT3 activation in MDSCs through toll-like receptor 2 (TLR2) [38]. Pre-metastatic cancer EVs induce immune surveillance by patrolling monocytes, which causes cancer cell clearance at the metastatic niche [41]. Moreover, levels of Programmed death-ligand 1 (PD-L1) carried by EVs correlate with patients’ disease activity, cancer stage, and lymph node [42].

Although tumor-derived EVs can escape immune surveillance, they can also trigger the cancer immune response [43]. EVs from heat-stressed tumor cells, which contain Hsp70, inhibit tumor growth by converting regulatory T cells to T helper 17 cells via Interleukin-6 (IL-6) [44]. EVs derived from a myeloid leukemia cell line, which is carrying cytokines, such as IL-15 and IL-18, activate NK cells and promote their cytotoxicity and proliferation [45]. Finally, treating an ex vivo culture of dendritic cells derived from the bone marrow with tumor-derived EVs promotes T-cell activation, supporting the role of cancer EVs in modulating the immune response [36].

Like other cancer-derived EVs, MM cell-derived EVs play a pivotal role in immune modulation [46,47]. BMSC-derived EVs activate the STAT3 pathway in MDSCs [48]. MM cell-derived EVs promote the viability and proliferation of MDSCs by up-regulating inducible nitric oxide [29]. MDSCs activated by EVs enhance the STAT3 pathway, which enhances the capacity for T-cell suppression. Another study showed that tumor-derived EVs stimulate the cytotoxic T-lymphocyte response [49]. The authors established three mouse myeloma cell lines engineered to secrete tumor necrosis factor-α (TNF-α), IL-2, and Interferon-γ (IFN-γ). EVs derived from the cell line engineered to secrete TNF-α more efficiently activate CD8+ T-cell responses. In summary, alteration of immune surveillance via EVs is important for MM progression; however, the involvement of cancer-derived EVs in the immune system in MM is not yet completely understood.

### 2.3. MM-Derived EVs in Osteoclast Activation

Osteolytic lesions are one of the key features of cancer in bone metastasis or cancer in bone, and this affect the quality of life of patients. Recent research revealed that osteolytic lesions are also mediated by EVs from cancer cells. Overexpression of miR-192 in EVs derived from highly metastatic lung cancer cells decreases osteolytic lesions in a mouse model [50]. Non-small cell lung cancer-derived EVs containing amphiregulin, a ligand of epidermal growth factor receptor (EGFR), activate the EGFR pathway in pre-osteoclasts, which in turn promotes the expression level of receptor activator of nuclear factor kappa-B ligand (RANKL) [51]. In addition to induction of osteoclast, inhibition of osteoblast is also a cause of bone lesions. Osteoclasts secrete miRNA-enriched EVs, by which miR-214 is transferred into osteoblasts to inhibit their function [52]. Further, miR-940 secreted from cancer cells induce an osteoblastic phenotype in the bone metastatic microenvironment by targeting ARHGAP1 and FAM134A [53].

MM cell-derived EVs positively modulate pre-osteoclast migration by increasing CXC chemokine receptor type 4 expression and promoting survival [54]. Similar results are observed with EVs derived from plasma in MM patients. Information demonstrating EVs are associated with osteoclast induction has not been widely reported; however, there are some reports showing that osteoclast differentiation is associated with miRNAs. For instance, miR-21 inhibits STAT3, which mediates RANKL gene activation. Inhibition of miR-21 restores the RANKL/osteoprotegerin ratio in MM-derived BMSCs and impairs the resorbing activity of mature osteoclasts [55]. Activation of osteoclasts leads to the formation of bone lesions, which is the most frequent complication with MM. Although there are few data on miRNAs in MM-derived EVs associated with osteoclast differentiation, further research will likely uncover miRNAs involved in osteoclastogenesis, leading to therapeutic targets for the treatment of bone lesions in MM patients.

### 2.4. Tumor-Derived EVs Mediate Drug Resistance

Drug resistance is a major problem in cancer treatment. Tumor-derived EVs are tightly involved in drug resistance in various types of cancer [56,57]. For instance, EVs derived from imatinib-resistant chronic myeloid leukemia cells mediate the horizontal transfer of a drug resistance trait by delivering miR-365 [56]. EV-mediated immune evasion in cancer cells is associated with human epidermal growth factor receptor (HER2)-targeted anticancer drug resistance [58]. Further, miR-1246 in EVs from breast cancer promotes the acquisition of drug resistance [59]. miR-1246, which is highly expressed in metastatic breast cancer cells, suppresses the expression level of its target gene, Cyclin-G2. Treatment with EVs derived from metastatic breast cancer cells enhances the viability, migration, and chemotherapy resistance in cancer cells. Moreover, tumor-derived EVs are associated with drug resistance in ovarian cancer [60]. Cisplatin induces the release of EVs from ovarian cancer, which induces invasiveness and drug resistance in bystander cells that take up the EVs. This response is inhibited when EV uptake inhibitors are used. Finally, cisplatin-resistant lung cancer cell-derived EVs increase cisplatin resistance of recipient cells by delivering miR-100-5p in EVs [61]. As described above, EVs promote drug resistance acquisition by cancer cells in many ways. Therefore, inhibiting the secretion of EVs may prevent drug resistance acquisition and may result in a prolonged therapeutic effect. Thus, understanding the molecular mechanisms of drug resistance mediated by cancer-derived EV transfer is necessary for the development of novel strategies for successful cancer treatment.

In MM, drug resistance acquisition is also a big problem in patients. EVs are also associated with the acquisition of drug resistance in MM [62]. For instance, BMSC-derived EVs induce tumor cell resistance to bortezomib, which is one of the key drugs for MM treatment, by activating several survival pathways [63]. BMSC-derived EVs significantly increase cell viability of MM in the presence of bortezomib. Genotoxic stress modulates the release of EVs from MM cells capable of activating NK cell cytokine production [64]. The ability of anticancer chemotherapy to enhance the immunogenic potential of malignant cells mainly relies on the establishment of immunogenic cell death and the release of damage-associated molecular patterns (DAMPs). MM cell-derived EVs can stimulate IFN-γ production, but not the cytotoxic activity of NK cells, through a mechanism based on the activation of the nuclear factor-κ B pathway in a TLR2- and HSP70-dependent manner. The CD56high NK cell subset is more responsive to EV-induced IFN-γ production mediated by TLR2 engagement. Together, these findings suggest a novel synergistic mechanism between chemotherapy and antitumor innate immune responses based on the drug promotion of nanovesicles exposing DAMPs for innate receptors.

Beside EVs, the involvement of miRNAs in MM drug-resistance has been reported. Hao et al have shown that suppression of miR-15a and miR-16 expressions by the IL-6 secretion from BMSCs promoted drug-resistance in MM cells [65]. Furthermore, Jagannathan et al have revealed that loss of miR-29 family members increases Proteasome activator complex subunit 4 (PSME4) expression and PSME4 levels to increase proteasome activity and render cells therapeutically resistant to proteasome inhibitors [66]. From these results, the authors proposed that miRNAs may offer a therapeutic advantage for MM treatment by inhibiting proteasomes without inducing pro-survival autophagy. As shown above, miRNAs are one of the main cargo in EVs and there are some possibilities that these miRNAs, which we have introduced, can be involved in EVs from MM.

Given the reports above, EVs in MM may also be associated with resistance to other therapeutic drugs. More studies of drug resistance in MM are emerging research projects. 

## 3. Clinical Application of EV-Associated miRNAs as Biomarkers and EV-Targeting Therapies in MM Patients

Currently, a biopsy is required for definite diagnosis of many cancers. In MM, bone marrow aspiration and/or biopsy are necessary for definitive diagnosis. Biopsy imposes a heavy burden on patients, thus less invasive diagnostic methods are needed. miRNAs in the serum EVs from cancer cells have been observed [67]; therefore, EVs are expected to serve as new biomarkers. A recent study showed the prognostic role of circulating miRNAs in MM-derived EVs [68]. The authors obtained serum samples from 156 newly diagnosed MM patients and found 22 miRNAs with significantly lower levels in MM patients than in healthy donors. Several of these miRNAs, such as let-7b, let-7e, miR-106a, miR-106b, miR-155, miR-16, miR-17, miR-18a, and miR-20a, were identified as significant predictors for progression-free survival. However, only let-7b and miR-18a were significant predictors for overall survival. Thus, these two miRNAs in EVs were identified to be independent predictors for both progression-free and overall survival in MM. Six miRNAs (miR-26a-5p, miR-29c-3p, miR-30b-5p, miR-30c-5p, miR-193a-5p, and miR-331-3p) were significantly down-regulated in poor responders to lenalidomide with low-dose dexamethasone treatment [69]. Furthermore, down-regulation of several miRNAs, including miR-16-5p, miR-15a-5p, miR-20a-5p, and miR-17-5p, was observed in the patients with bortezomib resistance [70], thus circulating EVs carrying miRNAs may be used to predict drug resistance. In summary, miRNAs in EVs may be used as novel biomarkers in MM to help choose a therapeutic strategy. Further investigation will be crucial for the realization of this clinical potential.

**Figure 2:**
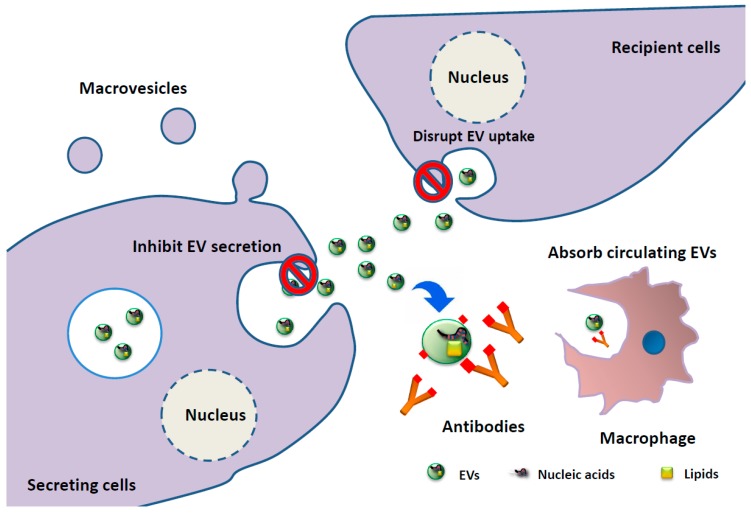
Strategy for extracellular vesicles (EV)-targeting therapy. Cancer-derived EVs have a crucial role in cancer development. Thus, EV elimination is potentially a new therapeutic strategy in MM. The first strategy is inhibition of EV secretion from MM cells. The second strategy is the absorption of circulating EVs. Recognition by EV-specific antibodies is followed by internalization by macrophages through phagocytosis. The third strategy is the disruption of EV uptake. These strategies have the potential to prevent cancer progression.

Another important issue is whether EVs from MM can be therapeutic targets (Figure 2). Recently, use of a monoclonal antibody, daratumumab (anti-CD38 antibody), drastically improved the prognosis of MM [71]. As mentioned above, cancer-derived EVs alter the cancer microenvironment, leading to cancer progression. Serum EVs express high levels of CD38 or CD138, which are considered markers of plasma cells and are often used as MM-related antigens [72,73,74]. Thus, eliminating EVs is a possible new therapeutic strategy in MM. Indeed, this strategy has already been tested. Antibodies against CD9 and CD63, which are enriched on EVs, were administrated to human breast cancer xenograft mouse models, and circulating administrated EVs tagged by anti-CD9 and -CD63 were internalized by macrophages through phagocytosis, resulting in the inhibition of cancer progression [75]. In addition to the neutralization of EVs by antibodies, inhibiting EV production from cancer cells is an attractive strategy for EV-targeting cancer therapy. HIFs and RAB22A mediate the formation of larger EVs and MVs, and stimulate breast cancer invasion and metastasis [21]. Exposure of human breast cancer cells to hypoxia augments MV shedding mediated by the HIF-dependent expression of the small GTPase Rab22A, which co-localizes with budding MVs at the cell surface. Incubation of naïve breast cancer cells with EVs shed by hypoxic breast cancer cells promotes focal adhesion formation, invasion, and metastasis. In addition to this, the neutral sphingomyelinase 2 regulates miR-210 secretion via EVs and induces angiogenesis, which is associated with metastasis [19]. These and other papers prompted us to hypothesize that attenuating the function of a molecule responsible for EV secretion could lead to the inhibition of EVs associated with cancer development. As we have mentioned, EVs in MM induce various alterations in the microenvironment. Although not many studies of EVs and miRNAs in EVs from MM have been reported, miRNAs in EVs are attractive as potential biomarkers and therapeutic targets for MM.

## 4. Conclusions

Here, we summarized the current knowledge of EVs and miRNAs in MM development and discussed the potential clinical applications of EVs as promising biomarkers and new therapeutic targets. EVs have huge possibility regarding the treatment of the disease in the variety of aspect. Recently, a drug delivery system using EVs loaded in target drugs in their lumen has been studied. Ohno et al demonstrated that EVs can efficiently deliver miRNA to EGFR-expressing breast cancer cells [76]. It has been shown that EVs can deliver a variety of bioactive cargos including small molecules [77], protein [78], and siRNA [79]. From these points of view, a drug delivery system using EVs is promising; however, further investigation is needed.

Two-thirds of patients with MM are older than 65, suggesting that aging might be related to the initiation of MM. MM is a progressive disease occurring via a pre-malignant condition clinically known as monoclonal gammopathy of undetermined significance (MGUS). It is already known that MGUS precedes MM and is associated with a risk of progression to MM [80]. Thus, early detection of MM is key to improving the outcome of patients with MM; however, repetitive diagnosis by computer tomography, magnetic resonance imaging, or biopsy is very expensive. In addition to the economical aspect, the quality of life of elderly patients must be considered. In this respect, miRNAs in EVs are ideal targets for diagnosis and therapy. As discussed above, circulating miRNAs in EVs can be detected in various cancers including MM, and these miRNAs could be good targets for cancer treatment. Although the involvement of EVs in MM initiation during aging has not yet been clarified, understanding the molecular mechanisms may lead to novel biomarkers, therapies, and prevention, and will, therefore, be of particular benefit to aging societies.

Further research is needed to translate these findings to a clinical setting; however, we believe that in the near future, EV-based treatments will render MM a curable disease.
